# Influence of a Th17-Inducing Cytokine Milieu on Phenotypical and Functional Properties of Regulatory T Cells in Chronic Inflammatory Arthritis

**DOI:** 10.3390/ijms26157339

**Published:** 2025-07-29

**Authors:** Tobias Schwarz, Giovanni Almanzar, Marie Wulfheide, Robert Woidich, Marie-Therese Holzer, Timotheos Christoforou, Leonie Karle, David Radtke, Franziska Brauneiser, Thomas Haaf, Ramya Potabattula, Gabriela Ortega, Klaus-Peter Lesch, Arne Schäfer, Sandrine Benoit, Astrid Schmieder, Matthias Goebeler, Marc Schmalzing, Martin Feuchtenberger, Martina Prelog

**Affiliations:** 1 Department of Pediatrics, Pediatric Rheumatology/Special Immunology, University Hospital Wuerzburg, 97080 Wuerzburg, Germany; mail@tobias-schwarz.net (T.S.); almanzar_g@ukw.de (G.A.); woidich_r@ukw.de (R.W.); m.holzer@uke.de (M.-T.H.);; 2 Department of Human Genetics, University of Wuerzburg, 97074 Wuerzburg, Germany; thomas.haaf@uni-wuerzburg.de (T.H.); ramya.potabattula@uni-wuerzburg.de (R.P.); 3 Department of Psychiatry, Division of Molecular Psychiatry, University Hospital Wuerzburg, 97080 Wuerzburg, Germany; ortega_g@ukw.de (G.O.); lesch_k@ukw.de (K.-P.L.); 4 Department of Internal Medicine II, Rheumatology/Clinical Immunology, University Hospital Wuerzburg, 97080 Wuerzburg, Germanyschmalzing_m@ukw.de (M.S.); martin.feuchtenberger@innklinikum.de (M.F.); 5 Department of Dermatology, Venereology and Allergology, University Hospital Wuerzburg, 97080 Wuerzburg, Germany; benoit_s@ukw.de (S.B.); schmieder_a@ukw.de (A.S.); goebeler_m1@ukw.de (M.G.); 6 Department of Rheumatology, Med|Bayern Ost Medizinische Versorgungszentren Burghausen-Altoetting GmbH, 84489 Burghausen, Germany

**Keywords:** Regulatory T cells, Th17 cells, FoxP3, methylation, TSDR, psoriatic arthritis

## Abstract

Considering the high plasticity of FoxP3^+^ regulatory T (Treg) cells and Interleukin (IL)-17-producing Th17 cells, we hypothesized that a Th17 inflammatory milieu may impair the functional properties of Treg cells in chronic inflammatory arthritides. Therefore, a cross-sectional explorative analysis was set up in patients with psoriatic arthritis (PsoA), rheumatoid arthritis, or spondyloarthritis to investigate the features of Th17 and Treg cells. T cell subpopulation counts, *FOXP3* mRNA expression, CpG methylation of the *FOXP3* gene, and the suppressive capacity of isolated Treg cells were determined. Ex vivo analysis of PsoA-derived peripheral blood lymphocytes showed a Th17-mediated inflammation. It was accompanied by demethylation of the *FOXP3* promotor and Treg-specific demethylated region (TSDR) in Treg cells which, however, resulted neither in elevated *FOXP3* mRNA expression nor in increased suppressive Treg cell capacity. To clarify this conundrum, in vitro stimulation of isolated Treg cells with Th17-inducing cytokines (IL-1β, IL-6, IL-23, TGFβ), recombinant IL-17, or the anti-IL-17A antibody secukinumab was performed, demonstrating that cell culture conditions polarizing towards Th17, but not IL-17 itself, impair the suppressive function of Treg cells, accompanied by diminished *FOXP3* mRNA expression due to hypermethylation of the *FOXP3* promotor and TSDR. This potential causal relationship between Th17 inflammation and impaired Treg cell function requires attention regarding the development of immunomodulatory therapies.

## 1. Introduction

CD4^+^ T helper (Th) cells, and among the Th subsets the interleukin (IL)-17-producing Th17 cells, play a crucial role in the progression of inflammation and organ failure in several chronic inflammatory and autoimmune diseases [1,2,3]. Regulatory T (Treg) cells, characterized by the expression of their master transcriptional regulator forkhead-box-P3 (FoxP3) [4], are essential for maintaining peripheral T cell homeostasis and tolerance, thereby preventing the development and aggravation of chronic inflammatory processes. T cell-mediated chronic inflammatory arthritides, such as spondyloarthritis (SpA) [5], psoriatic arthritis (PsoA) [6,7], and rheumatoid arthritis (RA) [8,9,10,11,12,13], have been associated with an imbalance between Treg and pro-inflammatory T cells [14].

Differentiation of naive Th0 into effector Th17 cells requires stimulation with IL-6 and TGFβ, resulting in the up-regulation of the transcription factors STAT3 and RORγ [15,16]. Pro-inflammatory cytokines, such as IL-1β and IL-23, enhance and stabilize the effector functions of Th17 cells [17,18]. However, in chronic inflammatory conditions, Th17 cells show marked plasticity, leading to the expression of the Th1 cell-associated cytokine interferon-γ (IFNγ), while losing the ability to produce IL-17 [19].

However, not only Th17 cells are able to change their secretome under chronic inflammatory conditions. Also, a high plasticity of Treg cells has been observed [20], resulting in a Treg-like phenotype lacking suppressive properties and acquiring functional features of pro-inflammatory T cells, resembling a Th17 phenotype [21,22,23,24]. Regarding the generation and maintenance of a stable Treg phenotype, demethylation of the *FOXP3* locus, particularly of the most relevant Treg-specific demethylated region (TSDR), is crucial [25,26,27,28,29,30]. Therefore, the reduced homeostatic properties of Treg cells under inflammatory pressure may be due to reduced FoxP3 expression, mediated by epigenetic modifications [31,32].

Considering the high plasticity of Treg cells, we hypothesized that the inflammatory cytokine milieu of a Th17 environment may impair the functional properties of Treg cells in Th17-associated chronic inflammatory arthritis. This is of particular relevance considering the available therapeutic monoclonal antibodies targeting the Th17-differentiation and effector pathways, in terms of antibodies directed against IL-23 or IL-17. Therefore, an explorative analysis was set up in patients with chronic inflammatory arthritides to investigate the features of Th17 and Treg cells with an emphasis on the functional capacity of isolated Treg cells in relation to the methylation status at the regulatory regions of the *FOXP3* locus.

## 2. Results

### 2.1. PsoA Patients Display Higher Proportions of Th17 Cells in the Peripheral Blood than RA or SpA Patients

In an initial exploratory analysis, we investigated the inflammatory and regulatory CD4^+^ T cell subsets in the peripheral blood of patients diagnosed with PsoA, RA, or SpA, compared to HCs. Sample size calculation was based on previous findings [24], resulting in a minimum number of seven participants per group (details in Section 4.7). Regarding the demographic and clinical characteristics of the study populations (Table 1), it should be noted that by matching the chronological age at the time of study, the PsoA and SpA patients had a longer disease duration than the RA patients due to diagnosis at an earlier age. Furthermore, despite ongoing therapy with biological disease-modifying anti-rheumatic drugs (bDMARDS), the PsoA patients displayed higher disease activity than the RA or SpA patients.

**Figure 1:**
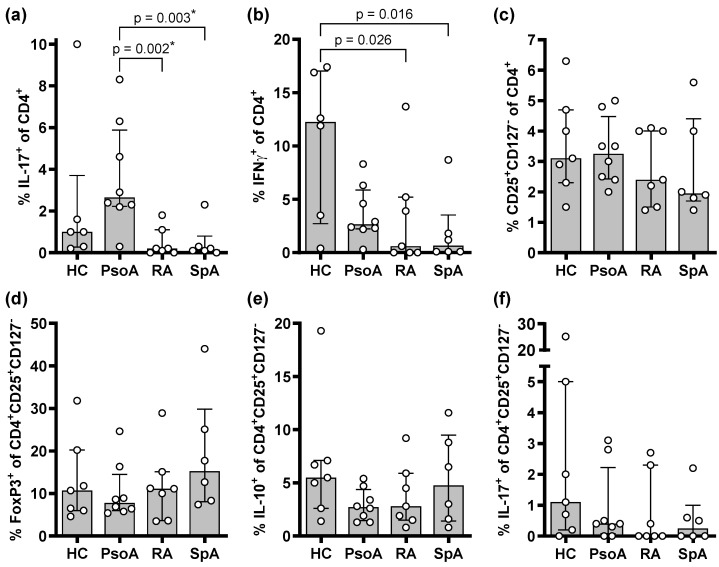
PsoA patients have elevated proportions of Th17 cells in the peripheral blood. The proportions of CD4^+^ Th cells expressing (**a**) interleukin (IL)-17 or (**b**) interferon (IFN)γ and the proportions of (**c**) CD25^+^CD127^−^ cells, as well as the proportions of CD4^+^CD25^+^CD127^−^ cells expressing (**d**) FoxP3, (**e**) IL-10, or (**f**) IL-17, were determined in the peripheral blood of HC subjects and PsoA, RA, and SpA patients using flow cytometry. Bars represent the median with the interquartile range, and open circles represent individual data. The Kruskal–Wallis test was performed, followed by Dunn’s test to compare the study populations. The two-stage step-up method of Benjamini, Krieger, and Yekutieli was applied to control the false discovery rate. Uncorrected *p*-values < 0.05 are depicted; asterisks indicate discoveries with a desired false discovery rate of Q < 0.05.

We performed flow cytometry to determine the relative proportions of helper T cells expressing the Th17-associated cytokine IL-17 and the Th1-associated cytokine IFNγ, as well as the proportions of CD4^+^CD25^+^CD127^−^ helper T cells expressing FoxP3, IL-10, or IL-17 (Figure 1). The PsoA patients demonstrated significantly higher peripheral proportions of IL-17-expressing helper T cells compared to the SpA or RA patients (Figure 1a). The proportions of IFNγ-positive Th cells tended to be lower in all chronic inflammatory arthritides, compared to the HCs (Figure 1b), whereas the proportions of CD4^+^CD25^+^CD127^dim/−^ T cells expressing FoxP3, IL-10, or IL-17 did not differ significantly between the arthritis forms or compared to the HCs (Figure 1c–f).

We also determined the concentrations of the cytokines IL-6, IL-10, IFNγ, TNFα, IL-17A, IL-17F, and IL-22 in serum samples of the arthritis patients (Supplementary Figure S1). Due to the low sample size of this initial exploratory analysis, no statistically significant differences were found. The PsoA as well as the SpA patients tended to have higher levels of IL-17A and IL-22, but also of TNFα than the RA patients.

### 2.2. Treg Cells of PsoA Patients Show Demethylation at the TSDR and Promotor Region of the FOXP3 Gene and Slightly Increased FOXP3 mRNA Expression Which, However, Is Not Associated with an Increase in Suppressive Capacity

**Figure 2:**
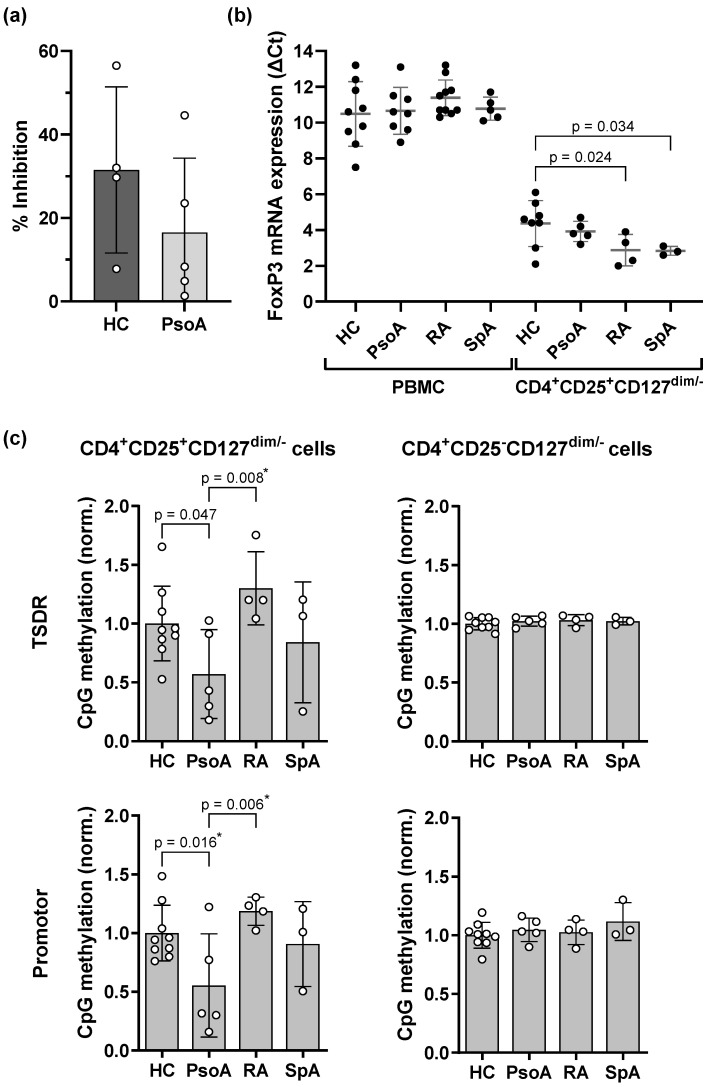
Suppressive function, *FOXP3* mRNA expression, and CpG methylation at the regulatory T (Treg)-specific demethylated region (TSDR) and promotor region of *FOXP3* in Treg cells of PsoA patients and HCs. (**a**) Effector cells from PsoA patients or HCs were labeled with carboxyfluorescein-succinimidyl-ester (CFSE) and co-cultured at ratios of 1:1 and 1:0 with isolated autologous CD4^+^CD25^+^CD127^dim/−^ Treg cells. Effector cell proliferation in terms of CFSE dilution was assessed by flow cytometry and the suppressive function of the Treg cells was calculated as the percentage inhibition in effector cell proliferation. (**b**) *FOXP3* mRNA expression was determined in peripheral blood mononuclear cells (PBMC) or isolated CD4^+^CD25^+^CD127^dim/−^ Treg cells of HCs and PsoA, RA, or SpA patients using quantitative real-time PCR (qPCR). (**c**) CpG methylation at the TSDR and promotor region of *FOXP3* was determined in CD4^+^CD25^+^CD127^dim/−^ Treg cells and CD4^+^CD25^−^CD127^dim/−^ T cells, isolated from PBMC of HCs and PsoA, RA, or SpA patients. The mean of the methylation of the individual CpGs within the respective region was calculated and normalized to the mean methylation of the respective male or female HCs. Bars represent the mean with standard deviation, and closed and open circles represent individual data. (**a**) An unpaired *t* test or (**b**,**c**) analysis of variance was performed to compare the study populations. The two-stage step-up method of Benjamini, Krieger, and Yekutieli was applied to control the false discovery rate. Uncorrected *p*-values < 0.05 are depicted; asterisks indicate discoveries with a desired false discovery rate of Q < 0.05.

Despite the fact that the proportions of Treg cells did not differ between the different arthritis forms and the HCs, we set out to further investigate the effect of the Th17 milieu in PsoA on Treg cell function. Treg cell suppression assays showed that the inhibitory function of isolated CD4^+^CD25^+^CD127^dim/−^ cells derived from the peripheral blood of PsoA patients was comparable to slightly reduced compared to Treg cells derived from HCs (Figure 2a). Furthermore, we analyzed the *FOXP3* mRNA expression in PBMC and isolated CD4^+^CD25^+^CD127^dim/−^ cells of HCs and chronic inflammatory arthritis patients by quantitative real-time PCR (qPCR, Figure 2b). In agreement with the proportions of FoxP3^+^CD25^+^CD127^−^ Th cells in the flow cytometric analysis (Figure 1d), the *FOXP3* mRNA expression in PBMC did not differ, neither between the chronic inflammatory arthritis forms nor in comparison to the HCs. However, the *FOXP3* mRNA expression in isolated CD4^+^CD25^+^CD127^dim/−^ T cells tended to be higher in the arthritis patients than in the HCs, with the highest expression levels in isolated cells of RA and SpA patients. As a stable Treg phenotype has been associated with demethylation of the *FOXP3* gene [25,26,27], we determined the CpG methylation levels at the TSDR, promotor, and enhancer of the *FOXP3* gene locus. Surprisingly, whereas *FOXP3* mRNA expression was highest in CD4^+^CD25^+^CD127^dim/−^ cells of RA and SpA patients (Figure 2b), methylation of the *FOXP3* TSDR and promotor was diminished in cells of PsoA patients only (Figure 2c). CpG methylation at the *FOXP3* TSDR and promotor region in CD4^+^CD25^−^CD127^dim/−^ cells, as well as the methylation levels at the *FOXP3* enhancer region (Supplementary Figure S2), did not differ between the chronic inflammatory arthritis groups or in comparison to the HCs.

### 2.3. Th17-Inducing Cytokines Impair the Suppressive Function and Reduce the FOXP3 mRNA Expression of Treg Cells

The divergence between a slightly reduced suppressive function of PsoA-derived Treg cells despite a hypomethylated *FOXP3* promotor and TSDR prompted us to refrain from further increasing the sample size and to perform experiments simulating an inflammatory milieu as known from inflamed joints in PsoA patients [33]. Therefore, we cultured CD4^+^CD25^+^CD127^dim/−^ cells, isolated from Pso(A) patients or HCs in a Th17-polarizing milieu (IL-1β, IL-6, IL-23, and TGFβ), as well as in the presence of excess recombinant IL-17 (rIL-17), the IL-17A-blocking antibody secukinumab, or a research-use-only IL-17A-blocking antibody. Sample size calculation was again based on previous findings [24].

**Figure 3:**
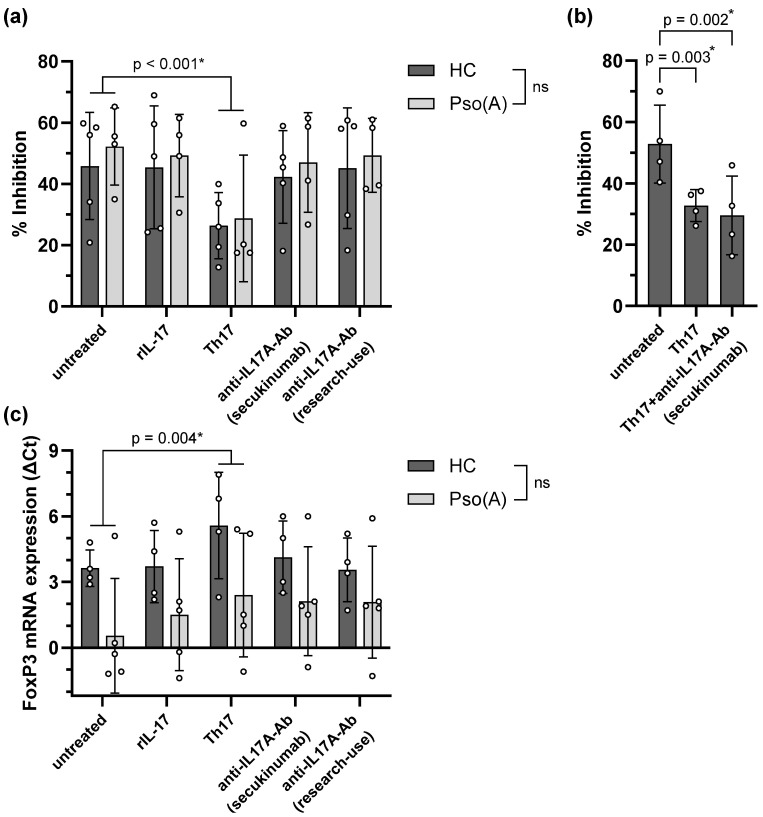
Suppressive function and *FOXP3* mRNA expression of Treg cells, in vitro stimulated with recombinant IL-17 (rIL-17), Th17-inducing cytokines (IL-1β, IL-6, IL-23, TGFβ), the anti-IL-17A antibody secukinumab, or a research-use-only anti-IL17A antibody. (**a**) CD4^+^CD25^+^CD127^dim/−^ cells were isolated from Pso(A) patients or HCs and incubated for 5 days, either with anti-CD3/CD28 antibodies alone (untreated), or in combination with rIL-17, Th17-inducing cytokines, or the respective anti-IL-17A antibodies. Following incubation, the Treg cells were washed and co-cultured for 5 days at ratios of 1:1 and 0:1 with CFSE-labeled autologous effector cells. Effector cell proliferation in terms of CFSE dilution was assessed by flow cytometry and the suppressive function of the Treg cells was calculated as the percentage inhibition in effector cell proliferation. (**b**) The Treg cell suppression assay with CD4^+^CD25^+^CD127^dim/−^ cells isolated from HCs was performed as in (**a**), following incubation with either anti-CD3/CD28 antibodies alone (untreated) or in combination with Th17-inducing cytokines, or in combination with Th17-inducing cytokines plus the anti-IL-17A antibody secukinumab. (**c**) mRNA expression of *FOXP3* was determined in CD4^+^CD25^+^CD127^dim/−^ cells isolated from Pso(A) patients or HCs, following incubation with anti-CD3/CD28 antibodies alone (untreated), or in combination with rIL-17, Th17-inducing cytokines, or the respective anti-IL-17A antibodies for 5 days. Bars represent the mean with standard deviation, and open circles represent individual data. (**a**,**c**) A two-way repeated measures analysis of variance including a possible interaction effect was performed to investigate the effects of the in vitro cell culture conditions and any difference between the Pso(A) patients and the HCs. (**b**) A repeated measures analysis of variance was performed. The two-stage step-up method of Benjamini, Krieger, and Yekutieli was applied to control the false discovery rate. Uncorrected *p*-values < 0.05 are depicted; asterisks indicate discoveries with a desired false discovery rate of Q < 0.05. ns = not significant.

The proportions of FoxP3^+^ and of Th17-like Treg cells did not change significantly due to culture of the CD4^+^CD25^+^CD127^dim/−^ cells under the varying conditions (Supplementary Figure S3). The Th17-inducing cytokines, however, significantly impaired the suppressive function of PsoA- as well as HC-derived Treg cells on autologous effector cells (Figure 3a). This effect was not due to IL-17 itself, as excess rIL-17 did not impair the suppressive function of Treg cells in vitro. This was corroborated by the fact that the diminished suppressive function of Th17-pulsed Treg cells could not be counteracted by concomitant treatment with the anti-IL17A antibody secukinumab (Figure 3b).

We also investigated the *FOXP3* mRNA expression following incubation of CD4^+^CD25^+^CD127^dim/−^ cells under Th17-inducing, excess-rIL-17, and IL-17-suppressed culture conditions (Figure 3c). Comparable to the ex vivo experiments (Figure 2b), *FOXP3* mRNA expression was slightly elevated in Pso(A)-derived CD4^+^CD25^+^CD127^dim/−^ T cells compared to the HCs, although the differences did not reach statistical significance. Moreover, consistent with the reduced Treg cell function, incubation of Treg cells in a Th17-inducing milieu diminished the expression of *FOXP3* mRNA, whereas incubation with excess rIL-17 did not have a relevant influence on the *FOXP3* mRNA expression.

### 2.4. Enhanced FOXP3 mRNA Expression in Pso(A)-Derived Treg Cells and Downregulated Expression Following Th17-Inducing Cytokine Conditions Are Mediated by an Altered Methylation of the TSDR and Promotor Region of the FOXP3 Gene

**Figure 4:**
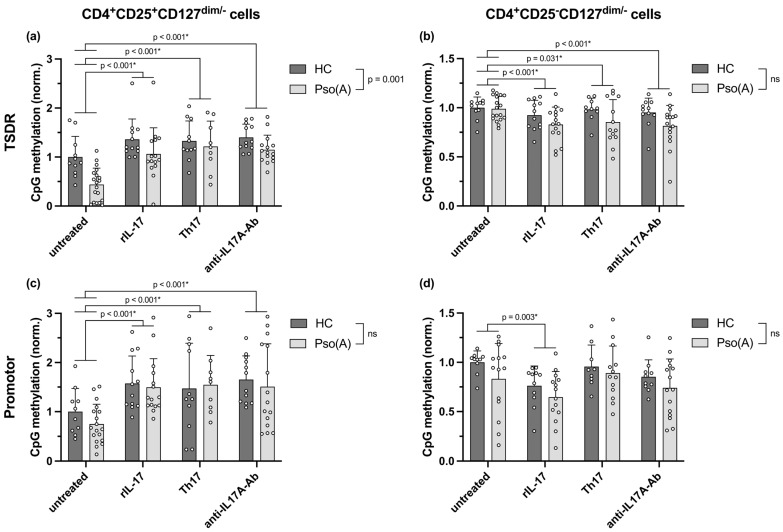
CpG methylation of the *FOXP3* TSDR and promotor region in Treg cells of Pso(A) patients and HCs, in vitro stimulated with rIL-17, Th17-inducing cytokines (IL-1β, IL-6, IL-23, TGFβ) or the anti-IL-17A antibody secukinumab. (**a**,**c**) CD4^+^CD25^+^CD127^dim/−^ and (**b**,**d**) CD4^+^CD25^−^CD127^dim/−^ cells were isolated from Pso(A) patients (*n* = 12) or HCs (*n* = 19) and incubated for 5 days, either with anti-CD3/CD28 antibodies alone (untreated) or in combination with rIL-17, Th17-inducing cytokines, or the anti-IL-17A antibody secukinumab. CpG methylation at the (**a**,**b**) TSDR and (**c**,**d**) promotor region of *FOXP3* was determined. The mean of the methylation of the individual CpGs within the respective region was calculated and normalized to the mean methylation of the respective male or female HCs. Bars represent the mean with standard deviation, and open circles represent individual data. Due to sporadic missing values for some culture conditions, a mixed-effects analysis of variance including a possible interaction effect was performed to investigate the effects of the in vitro cell culture conditions and any difference between the Pso(A) patients and the HCs. The two-stage step-up method of Benjamini, Krieger, and Yekutieli was applied to control the false discovery rate. Uncorrected *p*-values < 0.05 are depicted; asterisks indicate discoveries with a desired false discovery rate of Q < 0.05. ns = not significant.

In view of the observation that *FOXP3* mRNA expression in Treg cells is unaltered to slightly upregulated in Th17-associated Pso(A) (Figure 2b and Figure 3c) while Treg cells downregulate *FOXP3* mRNA expression under Th17-inducing cytokine conditions (Figure 3c), the methylation status following in vitro incubation of CD4^+^CD25^+^CD127^dim/−^ cells was assessed to understand the epigenetic mechanisms behind *FOXP3* mRNA expression in Treg cells of Pso(A) patients. Sample size calculation was based on the effect size regarding the differences in TSDR methylation in the ex vivo analysis of patients with differing inflammatory arthritides (Figure 2c), resulting in a total number of 22 subjects (details in Section 4.7). In agreement with the slightly elevated *FOXP3* mRNA expression in PsoA and the ex vivo methylation data (Figure 2c), TSDR, and—to a lesser extent—promotor hypomethylation were found in Pso(A) patients, especially in the untreated condition (Figure 4a,c). Surprisingly, however, not only the Th17-polarizing cytokine conditions but also an excess stimulation with rIL-17 as well as an IL-17 inhibition induced an augmented methylation of the TSDR and promotor region of *FOXP3* in Treg cells. Furthermore, all culture conditions reduced the CpG methylation of the *FOXP3* TSDR in CD4^+^CD25^−^CD127^dim/−^ cells, especially in the Pso(A) patients (Figure 4b), whereas the promotor region was primarily demethylated upon stimulation with rIL-17 (Figure 4d). CpG methylation of the enhancer region of *FOXP3* was not altered due to the differing in vitro cell culture conditions (Supplementary Figure S4).

To further verify the methylation data, the correlation of the methylation of the individual CpGs with each other and with the *FOXP3* mRNA expression in CD4^+^CD25^+^CD127^dim/−^ cells was determined (Supplementary Figure S5), showing that the methylation of the CpGs within the TSDR and promotor region correlated well with each other, whereas the CpG methylation of the *FOXP3* enhancer region did not correlate with the CpG methylation within the TSDR or promotor region. Furthermore, correlation analysis showed that the methylation of all CpGs within the TSDR and of two CpGs within the promotor region correlated significantly with the *FOXP3* mRNA expression, indicating a major influence of the TSDR on the *FOXP3* mRNA expression and a subordinate contribution of the promotor region.

## 3. Discussion

Considering the high plasticity of Treg cells and Th17 cells in chronic inflammatory conditions, we hypothesized that modulation of the cytokine milieu towards a Th17 environment may impair the functional properties of Treg cells in chronic inflammatory arthritis. In accordance with our hypothesis, we found that upon in vitro culture of Pso(A)- or HC-derived CD4^+^CD25^+^CD127^dim/−^ cells in a Th17-inducing cytokine milieu, the Treg cells displayed a diminished functional capacity (Figure 3a), associated with a reduced expression of *FOXP3* mRNA (Figure 3c) and an increased methylation of the *FOXP3* promotor and TSDR (Figure 4a,c). The decreased functional capacity of the Treg cells in a cytokine milieu polarizing towards Th17 could neither be induced by excess rIL-17 nor could it be restored by the addition of the anti-IL-17A antibody secukinumab to the Th17-inducing cytokine cocktail (Figure 3b), suggesting that the inhibitory effect on the Treg cells was mediated by the Th17-inducing cytokine milieu or Th17-associated cytokines like IL-21 or IL-22, but not by IL-17 alone. That inhibiting IL-17A does not markedly influence Treg cells is in agreement with gene expression profiling data of lesional T cell subsets from patients with psoriatic plaques treated with secukinumab [34]. It can be assumed that the cytokines used in the Th17-inducing cell culture are directly involved in the methylation of *FOXP3*. Particularly, high IL-6 and moderate to high TGFβ concentrations have been shown to influence *FOXP3* methylation, being associated with a rather unstable FoxP3 phenotype [35,36,37]. This implies that treatments inhibiting IL-6, IL-23, or IL1β signaling might be superior to IL-17 inhibitors in restoring Treg function in PsoA, RA, or SpA. Similarly, anti-TNFα therapy has been shown to reverse compromised Treg function in rheumatoid arthritis [38], and tyrosine kinase 2 (TYK2) inhibitors might prove superior to Janus kinase 1 (JAK1) inhibitors in the treatment of PsoA regarding Treg function [39]. However, further studies will be required to elucidate the influence of differing treatment regimens on Treg numbers and function.

The in vitro effects of the Th17-inducing cytokines were not specific for Treg cells of Pso(A) patients, even though the influence on the *FOXP3* methylation seemed to be slightly more pronounced within the Pso(A) group than the HC group. It currently remains unclear why incubation with excess rIL-17 or secukinumab also induced an increased methylation of the *FOXP3* promotor and TSDR in Treg cells. It may indicate that secondary, possibly autocrine, mechanisms might dynamically regulate IL-17 receptor molecules which may alter the selective influence of blockade or stimulation of Th17 pathways. In these cases, however, neither a reduced *FOXP3* expression nor a reduced functional capacity of the Treg cells was observed.

When we try to align our in vitro observations with the ex vivo data of patients with chronic inflammatory arthritides, PsoA is well known to be mediated by a Th17-type inflammatory response [6], an issue also found in our patient cohort (Figure 1a). Furthermore, the Treg cells of our PsoA patients displayed a tendency towards a diminished suppressive capacity (Figure 2a), suggesting that this might be mediated by the Th17-type inflammatory milieu in these patients. However, in contrast to the in vitro findings that a Th17-inducing cytokine milieu reduces the expression of FoxP3 in Treg cells (Figure 3c), the Treg cells isolated ex vivo displayed an unaltered to slightly elevated *FOXP3* mRNA expression (Figure 2b), associated with hypomethylation of the *FOXP3* promotor and TSDR (Figure 2c). The elevated *FOXP3* mRNA expression and diminished *FOXP3* methylation in Treg cells of PsoA patients compared to HCs ex vivo were also evident following in vitro culture with anti-CD3/CD28 antibodies without further stimulation (Figure 3c and Figure 4, ‘untreated’ control). A possible explanation for the discrepancy between the functional impairment of Treg cells in PsoA on the one hand and the *FOXP3* hypomethylation and increased mRNA levels on the other hand could be that a significant proportion of the isolated CD4^+^CD25^+^CD127^dim/−^ cells might not represent functional Treg cells. In the in vitro assays, the cytokine milieu polarizing towards Th17 not only induced methylation of the *FOXP3* TSDR and promotor in CD4^+^CD25^+^CD127^dim/−^ cells (Figure 4a), but also demethylation of the TSDR in CD4^+^CD25^−^CD127^dim/−^ cells (Figure 4b). It is therefore possible that the Th17-inducing cytokine milieu in PsoA patients induces demethylation of the *FOXP3* TSDR in effector T cells, thus enhancing *FOXP3* and CD25 expression without turning these cells into functional Treg cells.

It needs to be noted that for the in vitro stimulation experiments, Treg cells were isolated from Pso(A) patients with low disease activity or in stable remission, whereas the ex vivo data on PsoA was essentially gathered from patients with significant disease activity (Table 1). As autologous effector cells were used in the suppression assays, it is possible that the slightly reduced suppressive capacity of the PsoA-derived Treg cells (Figure 2a) is due to effector cell resistance rather than reduced Treg cell function. The inflammatory activity of the chronic arthritides might also explain why the lymphocyte subpopulations and *FOXP3* methylation of the RA and SpA patients were comparable to the HCs, as several studies indicated that the activity of chronic arthritides affects the Treg/Th17 balance and *FOXP3* methylation [40,41]. Along that line, Huang et al. [40] found an altered Treg/Th17 ratio and a hypermethylated *FOXP3* TSDR in early active or advanced active RA only, not in RA in stable remission. It needs to be noted, however, that the hypermethylated TSDR in early active RA patients in the latter study was determined in total PBMC, whereas the hypomethylated TSDR in active PsoA patients in our study was determined in isolated Treg cells. In our in vitro experiments, the cytokine milieus had opposing effects on CD4^+^CD25^+^CD127^dim/−^ and CD4^+^CD25^−^CD127^dim/−^ cells regarding the promotor and TSDR methylation (Figure 4). It therefore cannot be ruled out that the RA-associated alterations in *FOXP3* methylation determined by Huang et al. might primarily reflect non-Treg cells. Furthermore, it needs to be considered that FoxP3 is not only a marker for helper T cells with Treg features but is also expressed by activated non-Treg cells, which may significantly contribute to the peripheral T cell pool [42].

The results are limited by the cross-sectional study design and the relatively low number of participants included in the different disease and treatment arms. Furthermore, the patients in our study were not therapy-naive but had an advanced disease and were on a stable treatment regimen, in many cases with bDMARDs (Table 1). The differing treatment regimens and disease activities might have had a significant impact on the parameters measured, possibly contributing to the considerable variability in some of the data. Therefore, studies with larger cohorts will be required to investigate Treg cell function and *FOXP3* methylation in therapy-naive patients and patients on different treatment arms. Furthermore, in flow cytometry, absolute cell counts for some of the Th cell populations were rather low, thus contributing to the high variability of the data and the fact that we hardly found any significant differences between the patient cohorts in the flow cytometric analyses. Nevertheless, consistency between expression and methylation data underlines the accuracy of the in vitro data regarding the FoxP3 regulation under Th17-inducing cytokine conditions. But lymphocytes from peripheral blood and cell culture experiments may not be representative of the inflammatory situation in active joints or skin lesions, thus explaining the differences in the *FOXP3* expression and methylation between a Th17-inducing cell culture condition and a Th17-mediated disease entity.

## 4. Materials and Methods

### 4.1. Study Population

In a cross-sectional study design, peripheral venous blood was collected from Pso(A), SpA, and RA patients at the Department of Internal Medicine II (Division for Rheumatology/Clinical Immunology) and the Department of Dermatology, Venereology, and Allergology of the University Hospital Wuerzburg, as well as the Medical Care Center for Rheumatology, Burghausen, Germany, and was compared to blood samples from immunologically healthy controls (HCs) (Table 1). Exclusion criteria were inflammatory monogenetic syndromes, a history of malignoma, allergy or infections requiring medical attendance or vaccinations in the past 4 weeks. All participants gave their informed consent. The study was approved by the local ethics committee (protocol number 239/10) and conducted according to the principles of the Declaration of Helsinki 2013.

### 4.2. Flow Cytometry Analysis

Peripheral blood mononuclear cells (PBMC) were separated from freshly obtained blood by density gradient centrifugation according to standardized laboratory protocols and stored until use. The following T cell subpopulations were characterized by their expression of cell surface markers (all fluorochrome-labeled antibodies were purchased from BioLegend, San Diego, CA, USA): CD4 (APCFire or FITC) was used to identify helper T cells (Th), regulatory T cells (Treg) were defined as CD25bright (PECy7) and CD127-negative (BV421), and the chemokine receptor CCR6 (APC) was used to estimate the migratory ability of Th17 and Treg cells. Intracellular production of IFNγ (PE), IL-10 (APC), and IL-17A (BV421) (BioLegend) as well as of the Treg activation-characteristic transcription factor FoxP3 (PE) (BD Biosciences, San Jose, CA, USA) was determined following PMA/Ionomycin stimulation (Sigma-Aldrich, St. Louis, MO, USA) in the presence of brefeldin A as described [23]. Zombie dye (BioLegend) was used to exclude cell death. Serum cytokine concentrations of IL-6, IL-10, IFNγ, TNFα, IL-17A, IL-17F, and IL-22 were measured by LEGENDPlex^TM^ HU Th cytokine panel assay according to the manufacturer’s instructions (BioLegend). Flow cytometric analysis was performed using a FACSCanto II flow cytometer (BD Biosciences) and FACSDiva Software (Version 6.1.3, BD Biosciences). Representative dot plots from the flow cytometric analyses are shown in Supplementary Figure S6.

### 4.3. Isolation of Treg Cells and Cell Culture Conditions

CD4^+^CD25^+^CD127^dim/−^ T cells, significantly contributing to the peripheral Treg cell pool, were isolated using the MACS regulatory T cell isolation Kit II (Miltenyi Biotec, Bergisch Gladbach, Germany) according to the manufacturer’s protocol.

To assess the effects of a Th17-polarizing milieu, as well as a direct IL-17 stimulation and an IL-17 inhibition on Treg cells, isolated CD4^+^CD25^+^CD127^dim/−^ cells of PsoA patients and healthy controls were incubated for 5 days under varying culture conditions. To ensure T cell survival and activation, all cell cultures (including the ‘untreated’ control) were non-specifically stimulated with anti-CD3 (1 μg/mL) and anti-CD28 antibodies (0.5 μg/mL) (BioLegend). To imitate a Th17-polarizing milieu, a cocktail consisting of IL-1β (10 ng/mL), IL-6 (20 ng/mL), IL-23 (100 ng/mL), and TGFβ (5 ng/mL) (all purchased from BioLegend) was used according to pilot experiments [24]. Stimulation of Treg cells with rIL-17 (BioLegend) was conducted at a concentration of 50 ng/mL. For the inhibition of IL-17A, the monoclonal antibody secukinumab (Novartis, Basel, Switzerland) was used at a concentration of 10 μg/mL. To exclude unspecific or off-target effects of the therapeutic antibody secukinumab, a second monoclonal neutralizing research-use-only antibody against IL-17 (clone eBio64CAP17, Invitrogen Thermo Fisher, Carlsbad, CA, USA) was used at a concentration of 10 μg/mL.

### 4.4. Treg Cell Suppression Assay

To determine the suppressive capacity of the isolated Treg cells following incubation in the respective cytokine milieus, PBMC as effector cells (E) were labeled with carboxyfluorescein-succinimidyl-ester (CFSE, BioLegend), unspecifically stimulated with soluble anti-CD3/anti-CD28 antibodies (each 0.5 µg/mL), and co-cultured with the autologous Treg cells at specific ratios (E:Treg) of 1:0 and 1:1 for 5 days. Apart from the stimulation with anti-CD3/anti-CD28 antibodies, no stimulation with cytokines or inhibition with anti-IL17 antibodies was performed during the Treg suppression assay. CFSE dilution indicating proliferation was measured by flow cytometry. The suppressive capacity was calculated as

Inhibition [%] = (1 − (% proliferation (E:Treg 1:1)/% proliferation (E:Treg 1:0))) × 100.(1)

### 4.5. Quantitative Expression of FOXP3

*FOXP3* mRNA expression was determined by qPCR in PBMC and separated CD4^+^CD25^+^CD127^dim/−^ T cells as described [23]. RNA was extracted using the NucleoSpin RNA Mini kit (Marchery Nagel, Dueren, Germany). cDNA transcription was performed using the reverse Transcription Kit with RNase Inhibitor (Applied Biosystems, Darmstadt, Germany) and qPCR was performed with iTaq universal SYBR Green Supermix (Biorad, Ismaning, Germany) using an Applied Bio-systems^®^ Real-Time PCR7500 machine (Applied Biosystems, Darmstadt, Germany). The expression of *FOXP3* was normalized to the gene β2-microglobulin (*β2M*). For primers, see Supplementary Table S1 (all purchased from Eurofins Genomics, Luxembourg). Due to limited blood volume, qPCR could not be performed in all samples.

### 4.6. Bisulfite Pyrosequencing

The CpG methylation at the promoter, enhancer, and TSDR region of the *FOXP3* gene was investigated in DNA isolated from CD4^+^CD25^+^CD127^dim/−^ and CD4^+^CD25^−^CD127^dim/−^ T cells. We used the EpiTect96 Bisulfite Kit (Qiagen, Hilden, Germany) according to the manufacturer’s protocol for bisulfite conversion of DNA samples. Polymerase chain reaction (PCR) was performed as described elsewhere [43] and pyrosequencing was conducted on a PyroMark^®^ Q96 ID using the PyroMark Gold Q96 Reagents (Qiagen). Results were analyzed with the PyroMark Q96 Application Software (Version 2.5.10, Qiagen). Primers (Metabion international AG, Planegg, Germany, listed in Supplementary Tables S2 and S3) were designed with the PyroMark Assay Design Software (Version 2.0.2, Qiagen). Due to the X-chromosomal location of the *FOXP3* gene, CpG methylation differs between male and female patients. Therefore, the methylation levels were normalized to the mean methylation of the respective male and female HCs.

### 4.7. Statistical Analysis

Calculation of the sample size for the ex vivo analyses (Figure 1 and Figure 2) and the phenotypical and functional assays following in vitro stimulation of Treg cells (Figure 3) was based on previous findings [24]. Since in the cited publication in vitro incubation of Treg cells in a Th17-polarizing cytokine milieu had led to a reduction in the Treg cell suppressive capacity with an effect size of d = 1.7, sample size calculation resulted in a minimum number of 7 participants per group to achieve a power of 80% at a 5% level of significance. Sample size estimation for the epigenetic analyses following in vitro stimulation of Treg cells (Figure 4) was based on the effect size of η^2^ = 0.365 regarding the differences in the TSDR methylation in the ex vivo analyses of patients with differing inflammatory arthritides (Figure 2c). According to a mixed-effects analysis of variance with 2 groups and 4 conditions, sample size calculation resulted in a total number of 22 subjects to give us a power of 80% at a 5% level of significance.

Normal distribution of the data was evaluated by the Shapiro–Wilk test and assessment of the QQ-plot. To detect between-group differences with non-parametric data, the Mann–Whitney U test or the Kruskal–Wallis test, followed by Dunn’s multiple comparisons test, was performed. When data was normally distributed, F test or Brown–Forsythe test was used to ascertain equality of variances and an unpaired *t* test to compare two groups or analysis of variance to compare three or more groups was performed. To investigate the effects of the in vitro cell culture conditions on Treg cells of Pso(A) patients and HCs, a two-way repeated measures analysis of variance, or, in case of missing values, a mixed-effects analysis of variance including a possible interaction effect, was performed.

To adjust for multiple testing, the two-stage step-up method of Benjamini, Krieger, and Yekutieli [44] was applied to control the false discovery rate. Due to limited sample numbers, all uncorrected *p*-values < 0.05 are indicated in the graphs, but only those complying with a false discovery rate of Q < 0.05 are marked with an asterisk and were considered statistically significant.

Due to considerable variability in some of the data, the data were analyzed for outliers, in the case of non-parametric data defined as 1.5× the interquartile range below the 1st quartile or above the 3rd quartile, and in the case of parametric data defined as having a *z*-score < −2.58 or > 2.58. When outliers were identified, statistical analyses were carried out including and excluding them, but in no case was this associated with differing significance levels. The data and statistics shown include the outliers.

Pearson’s chi-square test or Fisher’s exact test was used for the comparison of categorical variables. Spearman rank correlation analyses were applied to assess the correlation of the methylation at the individual CpGs with each other and the *FOXP3* mRNA expression.

Statistical analyses were performed using SPSS (Version 29, IBM Corp., Armonk, NY, USA) and GraphPad Prism (Version 10.1, GraphPadSoftware, LLC, Boston, MA, USA).

## 5. Conclusions

A cell culture cytokine milieu polarizing differentiation of naive Th0 into effector Th17 cells induces epigenetic modifications at the TSDR and promotor region of *FOXP3*, resulting in a diminished FoxP3 expression and consequently Treg cell function on autologous effector cells, while IL-17 alone does not appear to be the major mediator of these effects. This finding is of significant importance, as secukinumab and other IL-17-targeting monoclonal antibodies are able to neutralize the biological function of IL-17 in PsoA, but according to our results, presumably are insufficient to neutralize the effects of a Th17-inducing cytokine milieu on Treg cell function.

## Figures and Tables

**Table 1:** Demographic and clinical characteristics of the study populations.

	Ex Vivo Phenotypical and Functional Assays (Figure 1 and Figure 2, Supplementary Figures S1 and S2)					Phenotypical and Functional Assays Following In Vitro Stimulation (Figure 3a,b, Supplementary Figure S3)			*FOXP3* Transcription and Methylation Following In Vitro Stimulation (Figure 3c and Figure 4, Supplementary Figures S4 and S5)		
	HC (*n* = 10)	PsoA (*n* = 8)	RA (*n* = 11)	SpA (*n* = 7)	*p*	HC (*n* = 8)	Pso(A) (*n* = 7)	*p*	HC (*n* = 12)	Pso(A) (*n* = 19)	*p*
Chronological age, median (range) years	52.5 (25–65)	54 (40–61)	59 (39–77)	58 (40–71)	0.263	23 (22–25)	50 (45–57)	**0.001**	27 (23–58)	46 (22–70)	**0.020**
Female sex, *n* (%)	7 (70)	3 (38)	8 (73)	3 (43)	0.306	2 (25)	3 (43)	0.608	6 (50)	6 (32)	0.452
Age at diagnosis, median (range) years	-	34 (16–55)	52 (34–75)	42 (29–58)	**0.025**	-	29 (13–44)	-	-	27 (5–62)	-
Disease duration, median (range) years	-	19.7 (3.7–45)	6.8 (1.3–25)	19.0 (1.3–25)	0.243	-	19 (3–36)	-	-	17 (5–28)	-
HLA-B27 positive/negative, *n* (%)	n.d.	3/2 (60/40)	3/3 (50/50)	3/3 (50/50)	0.932	n.d.	n.d.	-	n.d.	n.d.	-
RF positive/negative, *n* (%)	n.d.	n.d.	10/0 (100/0)	n.d.	-	n.d.	n.d.	-	n.d.	n.d.	-
CRP, median (range) mg/dL	n.d.	0.4 (0.0–3.1)	0.1 (0.0–0.5)	0.4 (0.1–1.4)	0.121	n.d.	0.2 (0.0–0.4)	-	n.d.	0.4 (0.1–2.0)	-
Blood leukocytes, median (range) × 10^9^/L	5.8 (4.5–8.3)	7.4 (6.2–10.1)	6.3 (4.9–13.6)	5.9 (5.1–9.0)	0.093	n.d.	7.4 (4.9–11.1)	-	n.d.	n.d.	-
Blood lymphocytes, median (range) × 10^9^/L	1.7 (1.5–2.3)	2.0 (1.4–3.0)	1.9 (0.6–2.6)	1.9 (1.3–2.1)	0.590	n.d.	1.6 (0.7–3.3)	-	n.d.	n.d.	-
Disease activity, NRS (0–10), median (range)	-	6.8 (3.0–8.5)	1.0 (0–6.0)	1.5 (0–3.0)	**0.008**	-	n.d.	-	-	n.d.	-
PASI, median (range)	-	n.d.	-	-	-	-	1.0 (0.5–5.8)	-	-	1.9 (0.0–4.6)	-
Psoriatic arthritis, *n* (%)	-	8 (100)	-	-	-	-	3 (43)	-	-	7 (37)	-
Treatment, *n* (%)											
None/local skin therapy only	-	0 (0)	0 (0)	0 (0)	-	-	0 (0)	-	-	2 (11)	-
Anti-IL-17 biologic agent	-	6 (75)	1 (9)	4 (57)	-	-	1 (14)	-	-	1 (5)	-
Anti-IL-12/23 biologic agent	-	2 (25)	0 (0)	0 (0)	-	-	2 (29)	-	-	9 (47)	-
Anti-TNF biologic agent	-	0 (0)	5 (45)	3 (43)	-	-	1 (14)	-	-	4 (21)	-
Janus kinase inhibitor	-	0 (0)	5 (45)	0 (0)	-	-	-	-	-	-	-
Methotrexate	-	1 (13)	3 (27)	1 (14)	-	-	3 (43)	-	-	2 (11)	-
Dimethyl fumarate	-	-	-	-	-	-	1 (14)	-	-	1 (5)	-
Apremilast	-	-	-	-	-	-	1 (14)	-	-	-	-
Systemic steroids	-	3 (38)	2 (18)	0 (0)	-	-	-	-	-	-	-
NSAID	-	5 (63)	2 (18)	1 (14)	-	-	-	-	-	-	-

Kruskal–Wallis test was performed for multiple comparisons, and Mann–Whitney U test was performed to compare the Pso(A) patients with the healthy controls. Pearson’s chi-square test or Fisher’s exact test was used for categorical variables. *p*-values < 0.05 are indicated in bold. HCs = healthy controls; PsoA = psoriatic arthritis patients; Pso(A) = patients with psoriasis or psoriatic arthritis; RA = rheumatoid arthritis patients; SpA = spondyloarthritis patients; n.d. = not determined; RF = rheumatoid factor; NRS = numeric rating scale; PASI = psoriasis area and severity index; TNF = tumor necrosis factor; NSAID = nonsteroidal anti-inflammatory drug; CRP = C-reactive protein.

## Data Availability

The original data presented in the study are included in the Supplementary Materials. Further inquiries can be directed to the corresponding author.

## Supplementary Materials

The supporting information can be downloaded at https://www.mdpi.com/article/10.3390/ijms26157339/s1.

## Abbreviations

The following abbreviations are used in this manuscript:

bDMARD	biological disease-modifying anti-rheumatic drug
β2M	β2-microglobulin
CFSE	carboxyfluorescein-succinimidyl-ester
CRP	C-reactive protein
E	effector cells
FoxP3	forkhead-box-P3
HCs	healthy controls
IFNγ	interferon-γ
IL	interleukin
JAK	Janus kinase
n.d.	not determined
NRS	numeric rating scale
ns	not significant
NSAID	nonsteroidal anti-inflammatory drug
PASI	psoriasis area and severity index
PBMC	peripheral blood mononuclear cells
PCR	polymerase chain reaction
PsoA	psoriatic arthritis
Pso(A)	psoriasis or psoriatic arthritis
qPCR	quantitative real-time PCR
RA	rheumatoid arthritis
RF	rheumatoid factor
rIL	recombinant interleukin
SpA	spondyloarthritis
Th	T helper
TNF	tumor necrosis factor
Treg	regulatory T
TSDR	Treg-specific demethylated region
TYK	Tyrosine kinase
