# Supplementary material for: Influence of a Th17-Inducing Cytokine Milieu on Phenotypical and Functional Properties of Regulatory T Cells in Chronic Inflammatory Arthritis
Source: Int J Mol Sci. 2025 Jul 29;26(15):7339. doi: 10.3390/ijms26157339 (PMC12347858; doi:10.3390/ijms26157339)
Supplement: Supplementary file 1 [file ijms-26-07339-s001.zip › suppl_information.v17.pdf]

International Journal of
Molecular Sciences

MDPI

Supplementary information: Influence of a Th17-inducing cytokine milieu on phenotypical and functional properties of regulatory T cells in chronic inflammatory arthritis

Tobias Schwarz, Giovanni Almanzar, Marie Wulfheide, Robert Woidich, Marie-Therese Holzer, Timotheos Christoforou, Leonie Karle, David Radtke, Franziska Brauneiser, Thomas Haaf, Ramya Potabattula, Gabriela Ortega, Klaus-Peter Lesch, Arne Schäfer, Sandrine Benoit, Astrid Schmieder, Matthias Goebeler, Marc Schmalzing, Martin Feuchtenberger and Martina Prelog

Academic Editor: Irmgard Tegeder and Cristina Belizna

Received: 20 April 2025
Revised: 25 July 2025
Accepted: 26 July 2025
Published: 29 July 2025

Citation: Schwarz, T.; Almanzar, G.; Wulfheide, M.; Woidich, R.; Holzer, M.-T.; Christoforou, T.; Karle, L.; Radtke, D.; Brauneiser, F.; Haaf, T.; et al. Influence of a Th17-Inducing Cytokine Milieu on Phenotypical and Functional Properties of Regulatory T Cells in Chronic Inflammatory Arthritis. *Int. J. Mol. Sci.* **2025**, *26*, 7339. https://doi.org/10.3390/ijms26157339

Copyright: © 2025 by the authors. Submitted for possible open access publication under the terms and conditions of the Creative Commons Attribution (CC BY) license (https://creativecommons.org/licenses/by/4.0/).

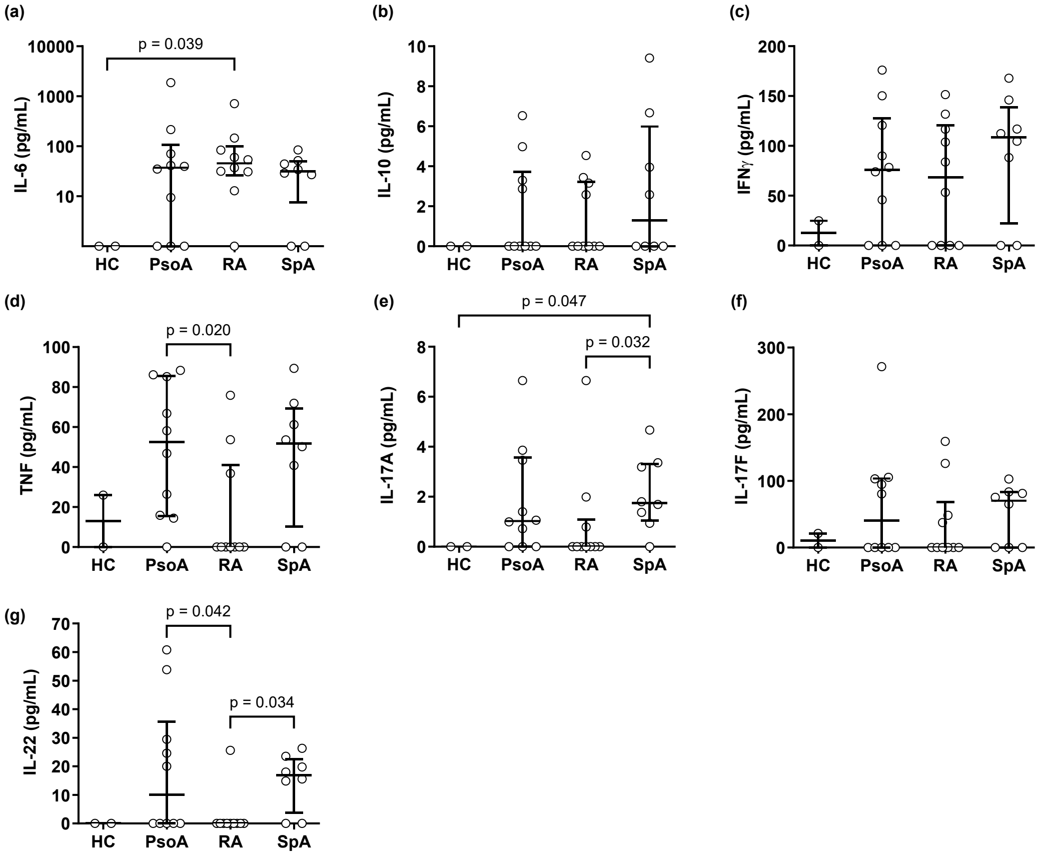

Figure S1. Serum cytokine levels in PsoA, RA, SpA patients and HC subjects. Cytokine concentrations of (**a**) IL-6, (**b**) IL-10, (**c**) IFNγ, (**d**) TNFα, (**e**) IL-17A, (**f**) IL-17F and (**g**) IL-22 were determined in serum samples of HC subjects and PsoA, RA and SpA patients by LegendPlex assay. Bars represent the median with the interquartile range, and open circles represent individual data. The Kruskal-Wallis test was performed, followed by Dunn's test to compare between the study populations. Uncorrected p-values < 0.05 are indicated. Following the two-stage step-up method of Benjamini, Krieger, and Yekutieli with a desired false discovery rate of Q < 0.05, these p-values were not flagged as discoveries, however.

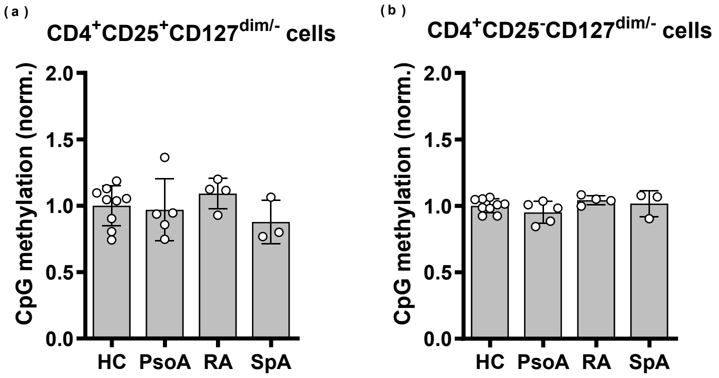

Figure S2: CpG methylation of the *FOXP3* enhancer region in Treg cells of PsoA, RA, SpA patients and HC subjects. CpG methylation at the enhancer region of *FOXP3* was determined in (**a**) $CD4^{+}CD25^{+}CD127^{dim/-}$ Treg cells and (**b**) $CD4^{+}CD25^{-}CD127^{dim/-}$ T cells, isolated from PBMC of HCs and PsoA, RA or SpA patients, using bisulfite pyrosequencing. The mean of the methylation of the individual CpGs within the enhancer region was calculated and normalized to the mean methylation of the respective male or female HCs. Bars represent the mean with standard deviation, and open circles represent individual data. Using analysis of variance, no statistically significant differences between the study groups were found.

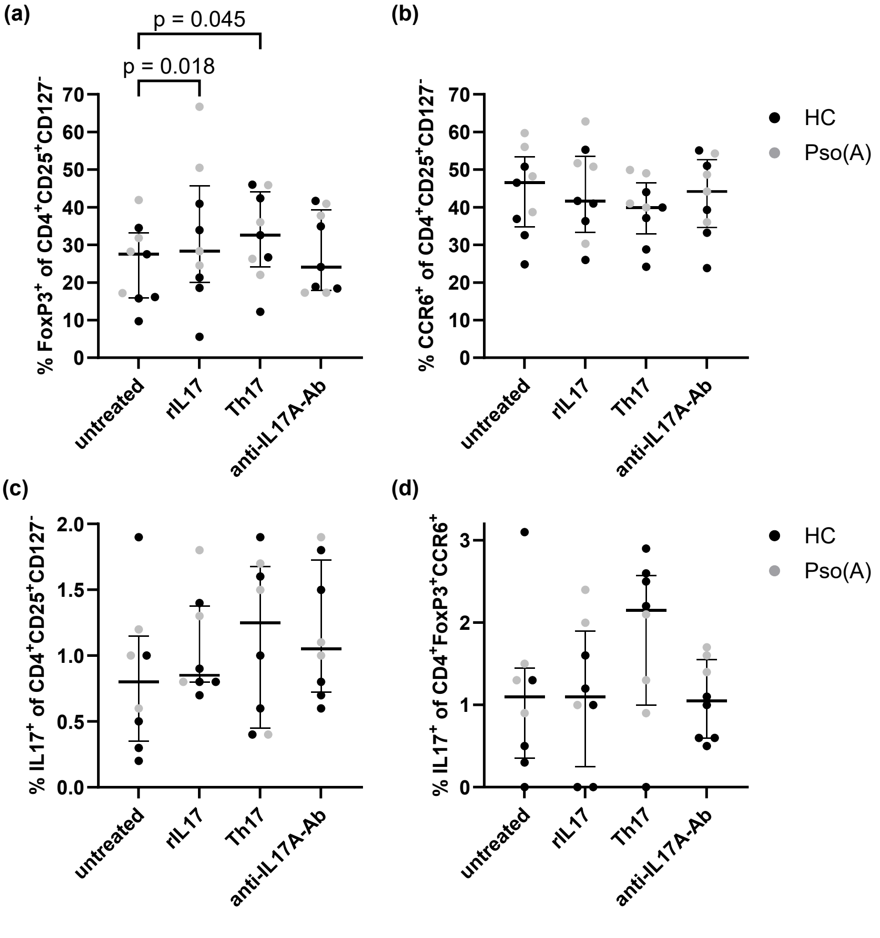

Figure S3: FoxP3, CCR6 and IL-17 expression by $CD4^{+}CD25^{+}CD127^{dim/-}$ Treg cells, in vitro stimulated with rIL-17, Th17-inducing cytokines or the anti-IL-17A antibody secukinumab. $CD4^{+}CD25^{+}CD127^{dim/-}$ cells were isolated from Pso(A) patients or HCs and incubated for 5 days, either with anti-CD3/CD28 antibodies alone (untreated), or in combination with rIL-17, Th17-inducing cytokines, or the anti-IL-17A antibody secukinumab. The proportions of

CD4$^+$CD25$^+$CD127$^-$ cells expressing (**a**) FoxP3, (**b**) CCR6, or (**c**) IL-17, and (**d**) the proportions of CD4$^+$FoxP3$^+$CCR6$^+$ cells expressing IL-17 were determined by flow cytometry. Bars represent the median with the interquartile range, and black and grey circles represent individual data of HCs and Pso(A) patients. The Friedmann test was performed to compare the individual culture conditions with the untreated Treg cells for the combined HCs and Pso(A)-patients' samples. Uncorrected *p*-values < 0.05 are indicated. Following the two-stage step-up method of Benjamini, Krieger, and Yekutieli with a desired false discovery rate of Q < 0.05, these *p*-values were not flagged as discoveries, however.

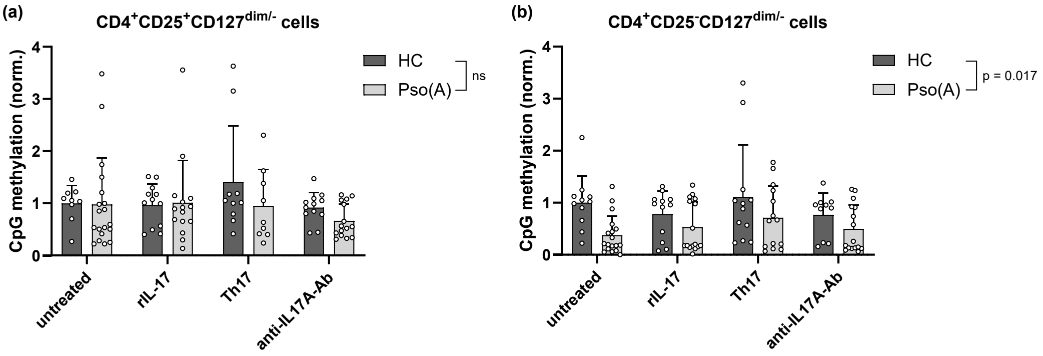

Figure S4: CpG methylation of the *FOXP3* enhancer region in Treg cells of Pso(A) patients and HCs, in vitro stimulated with rIL-17, Th17-inducing cytokines or the anti-IL-17A antibody secukinumab. (**a**) CD4$^+$CD25$^+$CD127$^{dim/-}$ and (**b**) CD4$^+$CD25$^-$CD127$^{dim/-}$ cells were isolated from Pso(A) patients (n=12) or HCs (n=19) and incubated for 5 days, either with anti-CD3/CD28 antibodies alone (untreated), or in combination with rIL-17, Th17-inducing cytokines or the anti-IL-17A antibody secukinumab. CpG methylation at the enhancer region of *FOXP3* was determined. The mean of the methylation of the individual CpGs within the enhancer region was calculated and normalized to the mean methylation of the respective male or female HCs. Bars represent the mean with standard deviation, and open circles represent individual data. Due to sporadic missing values for some culture conditions, a mixed-effects analysis including a possible interaction effect was performed to investigate the effects of the in vitro cell culture conditions and any possible difference between the Pso(A) patients and the HCs.

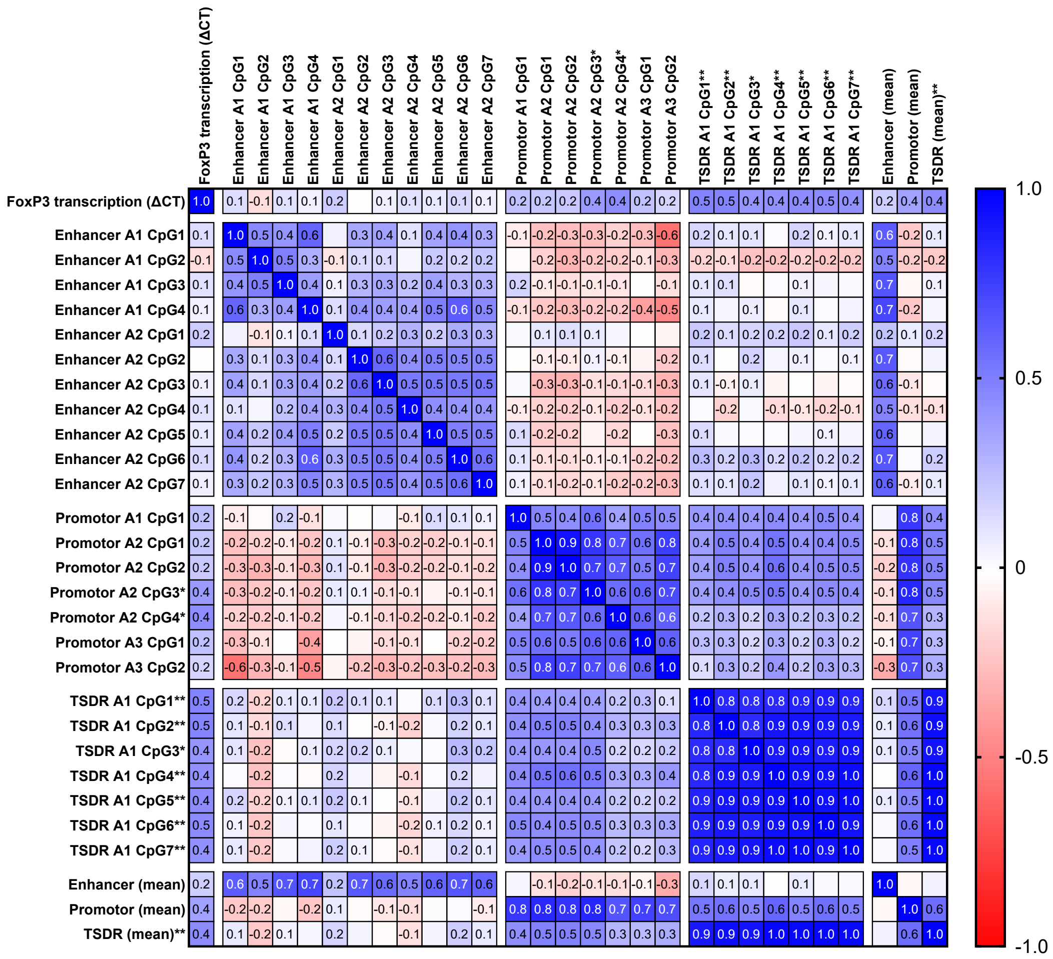

Figure S5: Correlation of the CpG methylation within the *FOXP3* enhancer, promotor and TSDR in CD4+CD25+CD127dim/- cells. Correlation matrix, depicting the spearman rank correlation coefficients between the normalized methylation of the individual CpGs within the enhancer, promotor and TSDR of *FOXP3*, the normalized mean of the methylation of the CpGs within the respective region, and the *FOXP3* mRNA expression. Data from CD4+CD25+CD127dim/- T cells isolated from Pso(A) patients or HCs, following in vitro culture under the various IL-17-stimulating/-inhibiting conditions, was pooled for the analysis. Where no correlation coefficient is indicated the coefficient is 0. Asterisks indicate statistically significant correlations between the particular CpG methylation and the *FOXP3* mRNA expression. The two-stage step-up method of Benjamini, Krieger, and Yekutieli was applied with a desired false discovery rate of Q < 0.05 to control the false discovery rate. Discoveries are indicated with * $p < 0.05$, ** $p < 0.01$.

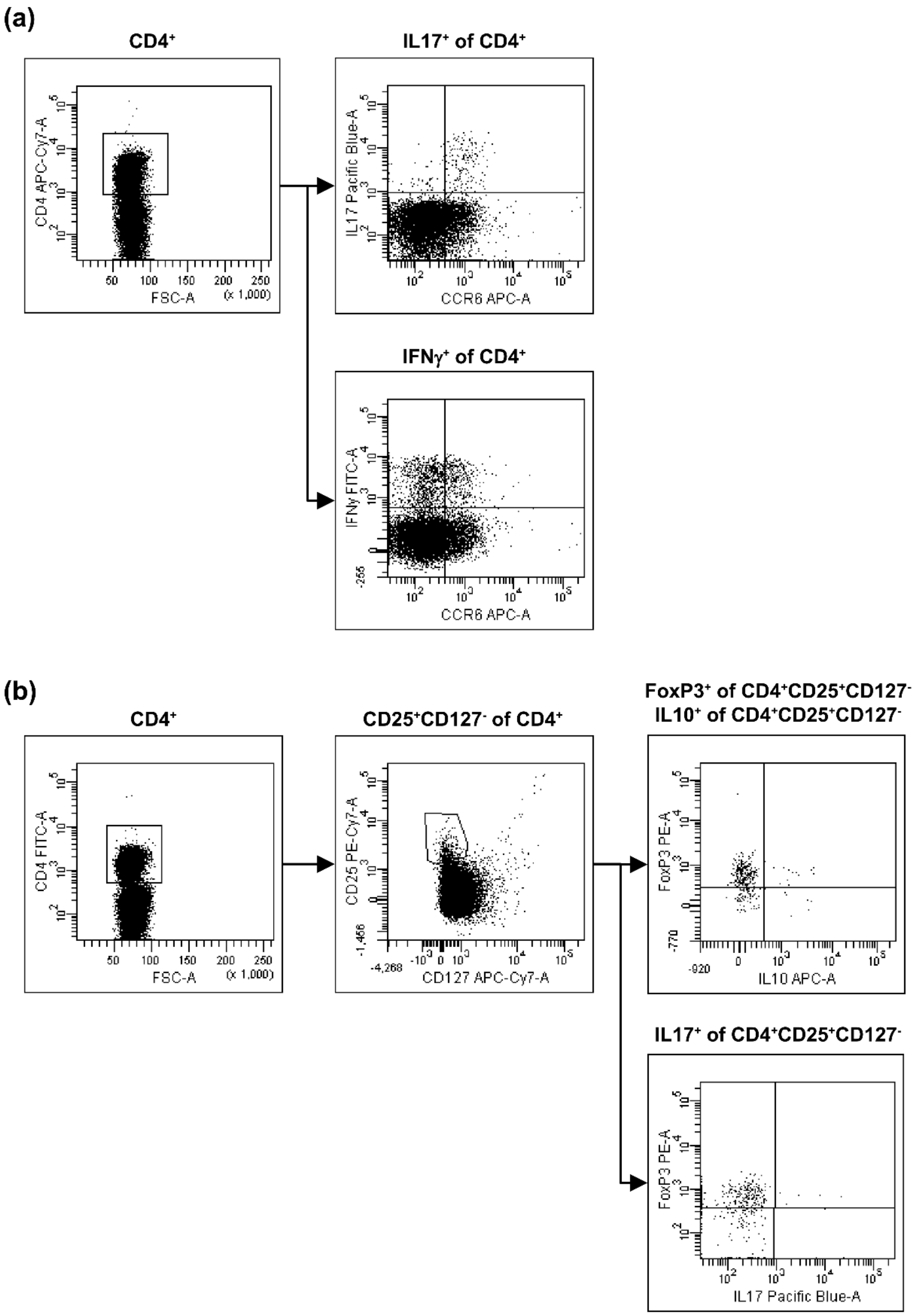
(a)
CD4⁺
IL17⁺ of CD4⁺
IFNγ⁺ of CD4⁺
CD4 APC-Cy7-A
FSC-A
(x 1,000)
IL17 Pacific Blue-A
CCR6 APC-A
IFNγ FITC-A
CCR6 APC-A
(b)
CD4⁺
CD25⁺CD127⁻ of CD4⁺
FoxP3⁺ of CD4⁺CD25⁺CD127⁻
IL10⁺ of CD4⁺CD25⁺CD127⁻
IL17⁺ of CD4⁺CD25⁺CD127⁻
CD4 FITC-A
FSC-A
(x 1,000)
CD25 PE-Cy7-A
CD127 APC-Cy7-A
FoxP3 PE-A
IL10 APC-A
FoxP3 PE-A
IL17 Pacific Blue-A

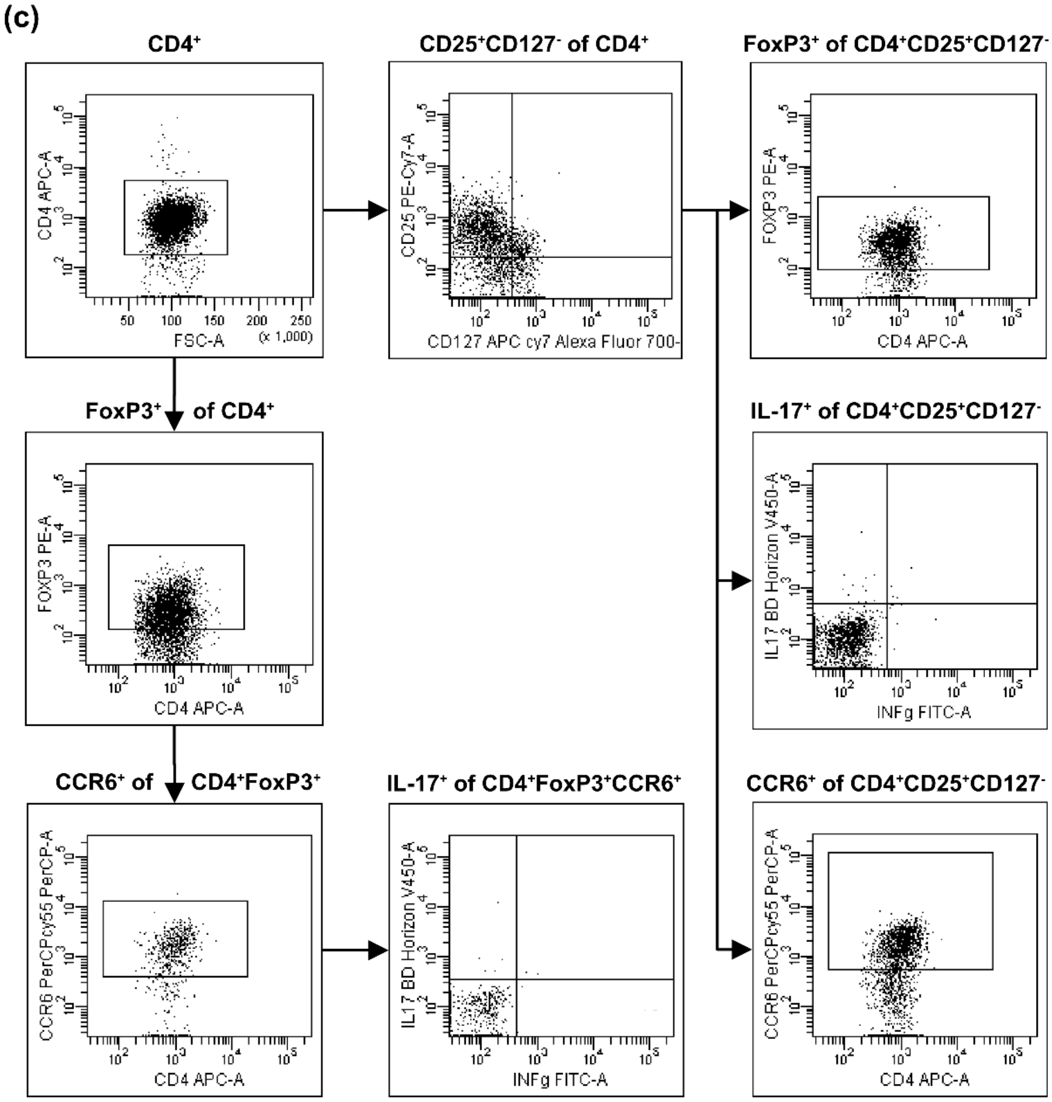

Figure S6: Gating strategy of the flow cytometric analyses performed. Peripheral blood mononuclear cells were separated from whole blood by density gradient centrifugation and the proportions of $CD4^+$ Th cells expressing (**a**) IL-17 or IFNγ, (**b**) the proportions of $CD25^+CD127^-$ cells, as well as the proportions of $CD4^+CD25^+CD127^-$ cells expressing FoxP3, IL-10 or IL-17 were determined. (**c**) $CD4^+CD25^+CD127^{dim/-}$ cells were isolated from PBMC using magnetic bead separation and incubated for 5 days with differing in vitro culture conditions. The proportions of $CD4^+CD25^+CD127^-$ cells expressing FoxP3, IL-17, or CCR6, as well as the proportions of $CD4^+FoxP3^+CCR6^+$ cells expressing IL-17 were determined.

Table S1. PCR primers used for quantitative real-time PCR.

	Forward sequence (5′-3′)	Reverse sequence (5′-3′)
FOXP3	TCA TCT GTG GCA TCA TCC GA	GGA ACT CTG GGA ATG TGC TG
β-2-microglobulin (*β2M*)	CCA GCA GAG AAT GGA AAG TC	GAT GCT GCT TAC ATG TCT CG

Table S2. PCR primers used for bisulfite converted DNA samples.

	Forward sequence (5′-3′)	Forward sequence (5′-3′)
FOXP3 TSDR	GTT TGT ATT TGG GTT TTG TTG TTA TAG TT	Biotin-AAA ATA TCT ACC CTC TTC TCT TCC
FOXP3 Enhancer Bis	ATG AAG GGG AGG AGG AAG	Biotin-CCT CCA ACT CCA CCA TAA
FOXP3 Promoter Bis	Biotin-AGT TTG GTT TGT GGG AAA TTG TT	ACC CTA TTA TCT CAT TAA TAC CTC TCA

Table S3. Primers used for pyrosequencing.

	Sequence (5′-3′)	Number of CpGs analyzed
FOXP3 TSDR Pyro	GGG TTT TGT TGT TAT AGT TT	7
FOXP3 Enhancer Bis Pyro 1	GAG GAA GAG GAG GTT	4
FOXP3 Enhancer Bis Pyro 2	GGG TTT TAT TTG GTT TTT ATA TT	7
FOXP3 Promoter Bis Pyro 1	ATA AAA ACA AAA TTA TTT TTA ATA	1
FOXP3 Promoter Bis Pyro 2	AAA TTA TTA AAA AAA AAA AAT CTA C	4
FOXP3 Promoter Bis Pyro 3	AAA AAC CCC CCC CCA	2

Disclaimer/Publisher's Note: The statements, opinions and data contained in all publications are solely those of the individual author(s) and contributor(s) and not of MDPI and/or the editor(s). MDPI and/or the editor(s) disclaim responsibility for any injury to people or property resulting from any ideas, methods, instructions or products referred to in the content.
